# A dual-switch safety system enables safety controllability and high-efficiency enrichment for T cell immunotherapy

**DOI:** 10.1016/j.omta.2026.201865

**Published:** 2026-10-14

**Authors:** Hongtao Liu, Huimin Wang, Bo Hou, Wenxin Zhou, Hanfei Zhu, Xin Lin, Jiasheng Wang

**Affiliations:** 1 Changping Laboratory, Yard 28, Science Park Road, Changping District, Beijing 102206, China; 2 School of Basic Medical Sciences, Tsinghua University, Beijing 100084, China; 3 Tsinghua-Peking Center for Life Sciences, Tsinghua University, Beijing 10084, China; 4 Department of Chemical Engineering, Tsinghua University, Beijing 100084, China; 5 Yifei (Hangzhou) Bio-tech Co., Ltd, Hangzhou 311305, China

**Keywords:** tHER2-TK, suicide switch, safety-controllable, enrichment, T cell immunotherapy

## Abstract

Chimeric antigen receptor (CAR) T cell therapy has achieved durable and complete responses in a substantial proportion of patients with B cell malignancies. However, during CAR-T cell therapy, the occurrence of adverse events—including on-target/off-tumor toxicity against healthy tissues, cytokine release syndrome (CRS), immune effector cell-associated neurotoxicity syndrome (ICANS), and the risk of T cell malignancies arising from random genomic integration of viral vectors—significantly threatens patient safety. In this study, we developed a novel truncated HER2 (tHER2)-herpes simplex virus thymidine kinase (HSVTK) dual-switch safety system and site-specifically integrated it into the *PDCD1* locus using a Cas12a/adeno-associated virus (AAV)-mediated delivery platform. Importantly, this dual-switch system enables efficient magnetic bead-based enrichment of tHER2-HSVTK (tHER2-TK)-positive T cells, allows tunable modulation of engineered T cell persistence through Herceptin or the small-molecule drug ganciclovir (GCV), and also provides a highly effective purification method before infusion. Collectively, these features enhance safety without compromising therapeutic efficacy, facilitating the development of safer and more controllable engineered T cell immunotherapies.

## Graphical abstract

**Figure:**
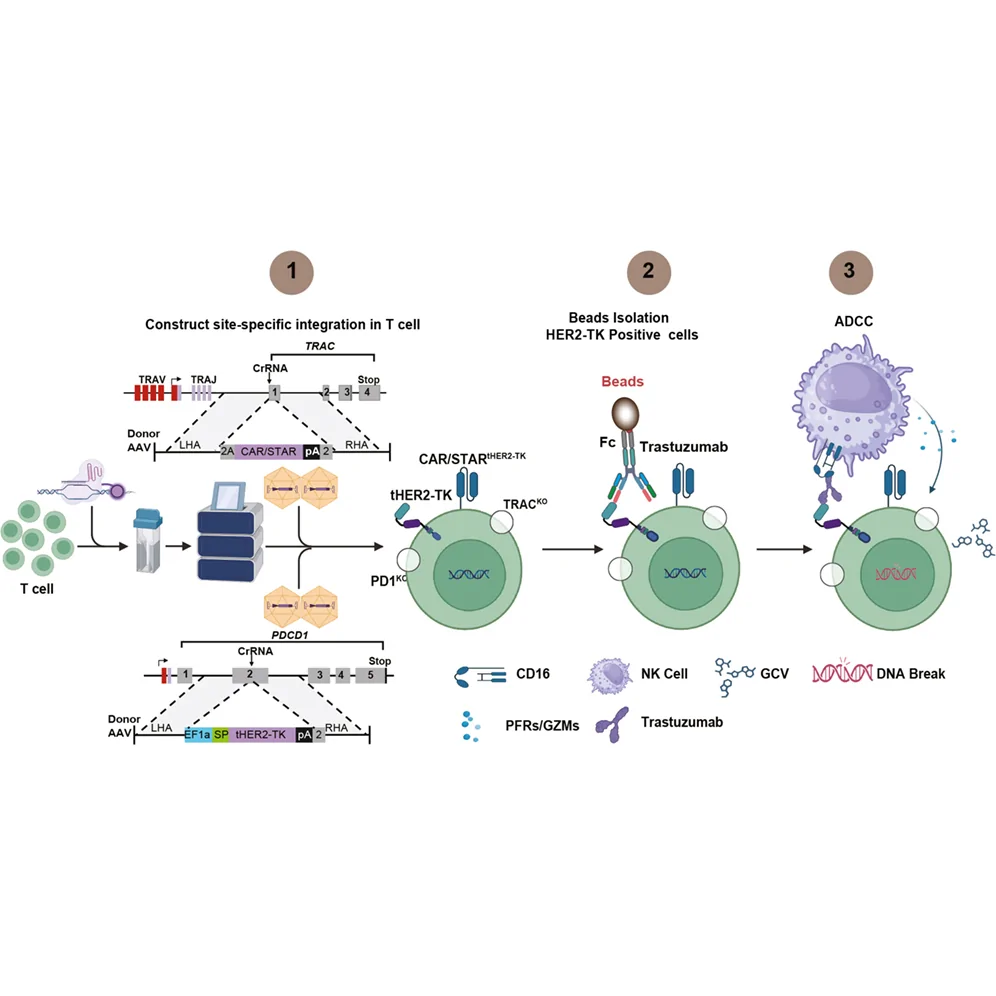

## Teaser

Wang and colleagues developed a dual-switch safety system (tHER2-HSVTK) enabling rapid antibody-mediated or gradual drug-induced elimination of engineered T cells, alongside magnetic bead-based enrichment. This strategy enhances the safety, controllability, and manufacturability of T cell immunotherapy while preserving therapeutic efficacy.

## Introduction

Adoptive T cell therapy (ACT), which includes engineered T cell receptor (TCR) and chimeric antigen receptor (CAR) T cell therapy, has demonstrated remarkable clinical potential in cancer treatment.^1^^,^^2^^,^^3^^,^^4^ Pioneering clinical trials using CAR-T cell successfully induced marked remission in patients with hematological malignancies, marked a turning point in ACT.^5^^,^^6^^,^^7^ Since the first Food and Drug Administration (FDA) approval in 2017, seven CAR-T therapies have been authorized for commercial use in treating leukemia, lymphomas, and multiple myeloma, with additional products also approved in China.^8^^,^^9^^,^^10^^,^^11^^,^^12^ However, the substantial therapeutic benefits of CAR-T cells are accompanied by notable safety concerns.^7^ In November 2023, the FDA issued a safety communication regarding reports of T cell malignancies, including CAR-positive lymphoma in patients who had received autologous CAR-T cell immunotherapies.^13^^,^^14^^,^^15^ However, subsequent real-world studies have indicated the incidence of such malignancies is only 0.03% with a 4-year cumulative incidence of 0.6%.^16^ The intrinsic oncogenic potential associated with random genomic integration of viral vector-mediated transgenes remains a significant concern, as this process may lead to uncontrolled T cell proliferation and even secondary hematological malignancies.^17^

Beyond the risk of secondary malignancies, CAR-T therapy can induce severe, life-threatening adverse effects, including cytokine release syndrome (CRS), graft-versus-host disease (GVHD), and immune effector cell-associated neurotoxicity syndrome (ICANS).^18^^,^^19^^,^^20^ Furthermore, the persistence of highly active engineered T cells may lead to on-target, off-tumor (OTOT) effects and damage to healthy tissues.^21^ These safety concerns underscore the urgent need for strategies to improve the controllability and safety of T cell immunotherapies. A promising approach involves the overexpression of safety and controllable systems within engineered T cells. For example, the inducible caspase-9 (iCasp9) and herpes simplex virus thymidine kinase (HSVTK) systems enable the selective ablation of engineered cells upon administration of a small-molecule activator.^22^^,^^23^^,^^24^^,^^25^^,^^26^^,^^27^ Alternatively, surface markers such as CD20 and EGFR can serve as targets for antibody-dependent cellular cytotoxicity (ADCC) medicated by rituximab or cetuximab.^28^^,^^29^^,^^30^^,^^31^^,^^32^ The truncated human EGFR (tEGFR)/cetuximab system represents one of the most clinically advanced antibody-dependent safety switches, having been evaluated in both preclinical models and clinical trials.^28^^,^^33^^,^^34^ However, antibody drugs targeting T cell-expressed surface markers may themselves induce OTOT toxicity. For instance, targeting tEGFR with cetuximab has been associated with toxic side effects primarily manifesting as dermatological and gastrointestinal reactions.^35^ Tyrosine kinase HER2 (also known as Neu, ErbB2), another member of EGFR family, is minimally expressed in normal tissues. The first anti-HER2 monoclonal antibody (mAb)—trastuzumab (also known as Herceptin)—can efficiently eliminate HER2-expressing tumor cells via ADCC.^22^^,^^36^^,^^37^^,^^38^ Trastuzumab is associated with well-documented cardiotoxicity such as arrhythmia, cardiomyopathy, and heart failure, which typically manifests as a reduced left ventricular ejection fraction (LVEF), but the exact pathogenesis of this event is unknown.^39^^,^^40^ However, these risks are established in the setting of chronic, repeated-dose trastuzumab administration for HER2-positive breast cancer therapy, typically administered weekly or every 3 weeks.^41^^,^^42^ Inspired by the potent ADCC effect induced by Herceptin to eliminate HER2-overexpressing tumor cells, we propose that leveraging this process could serve as a fast-responsive safety switch to control the number or persistence of adoptively transferred cells, thereby mitigating the risks and toxicities associated with ACT. Concurrently, we also propose utilizing this safety switch to achieve high-level enrichment of HER2-positive cells using magnetic beads. Furthermore, the HSVTK suicide gene system has demonstrated clinical utility as a safety switch, wherein administration of antiviral prodrug such as ganciclovir (GCV) inhibits DNA replication and selectively eliminates transgene-expressing cells during the therapeutic window.^24^ The use of HSVTK in adoptive T cell therapies has been extensively investigated, and its clinical applicability has been significantly limited by its high immunogenicity, as demonstrated in multiple clinical studies.^32^^,^^43^^,^^44^

To achieve efficient target cell elimination while maintaining safety and controllability, we developed a dual-target safety-switch system that integrates both the Herceptin/ADCC and HSVTK/GCV killing mechanism. This dual-target safety-switch system was site-specifically integrated into the *PDCD1* locus of adoptive T cells. Using this strategy avoids random genomic integration, ensures stable expression of the safety switch, facilitates easy isolation via magnetic beads, and enables precise intervention using either antibodies or small-molecule drugs at desired time points. By enabling safe, highly efficient, and selective control, this approach mitigates the risk of oncogenesis and off-tumor toxicities associated with allogeneic or autologous engineered cell immunotherapies.

## Results

### Targeting truncated HER2 to the *PDCD1* locus in human T cells

HER2 comprises an extracellular domain, a single transmembrane domain, and a cytoplasmic domain (total 1,255 amino acids). This receptor can be recognized by the FDA-approved antibody trastuzumab (Herceptin), which triggers natural killer (NK) cells to secrete perforins and granzymes, which induce tumor cell death.^39^ In this study, we constructed a truncated form of HER2 (tHER2) by fusing in tandem the extracellular domain Ⅲ, domain Ⅳ (which mediates high-affinity antibody binding), and the transmembrane domain (Figure 1A). The tHER2 expression cassette, driven by the exogenous EF1α promoter, was subsequently cloned into the recombinant adeno-associated virus serotype 6 (rAAV6). Additionally, we designed a CRISPR RNA (crRNA) targeting the 5′ end of the second exon of *PDCD1* to facilitate site-specific gene integration into the *PDCD1* locus (thereby reducing T cell exhaustion and taking advantage of this site’s permissiveness for integration) using the Cas12a/AAV delivery system (Figures 1B and 1C). Following electroporation with Cas12a and *PDCD1*-targeting Ribonucleoprotein (RNP), T cells were transduced with tHER2-encoding AAV virus at a multiplicity of infection (MOI) of 5 × 10^5^ (Figure 1D). After 4 days of culture and expansion, PCR analysis confirmed targeted integration of tHER2 into the *PDCD1* locus at the genomic level (Figure 1E), indicating that tHER2 was specifically inserted into the *PDCD1* gene. Flow cytometry results further demonstrated stable expression of tHER2 on T cells derived from different human donors, with an integration and expression efficiencies ranging from approximately 50% (Figures 1F and 1G); the gating strategy is shown in Figure S5A. Collectively, these results indicate that tHER2 was successfully integrated into the *PDCD1* locus and stably expressed at high efficiency in human primary T cells.

**Figure 1:**
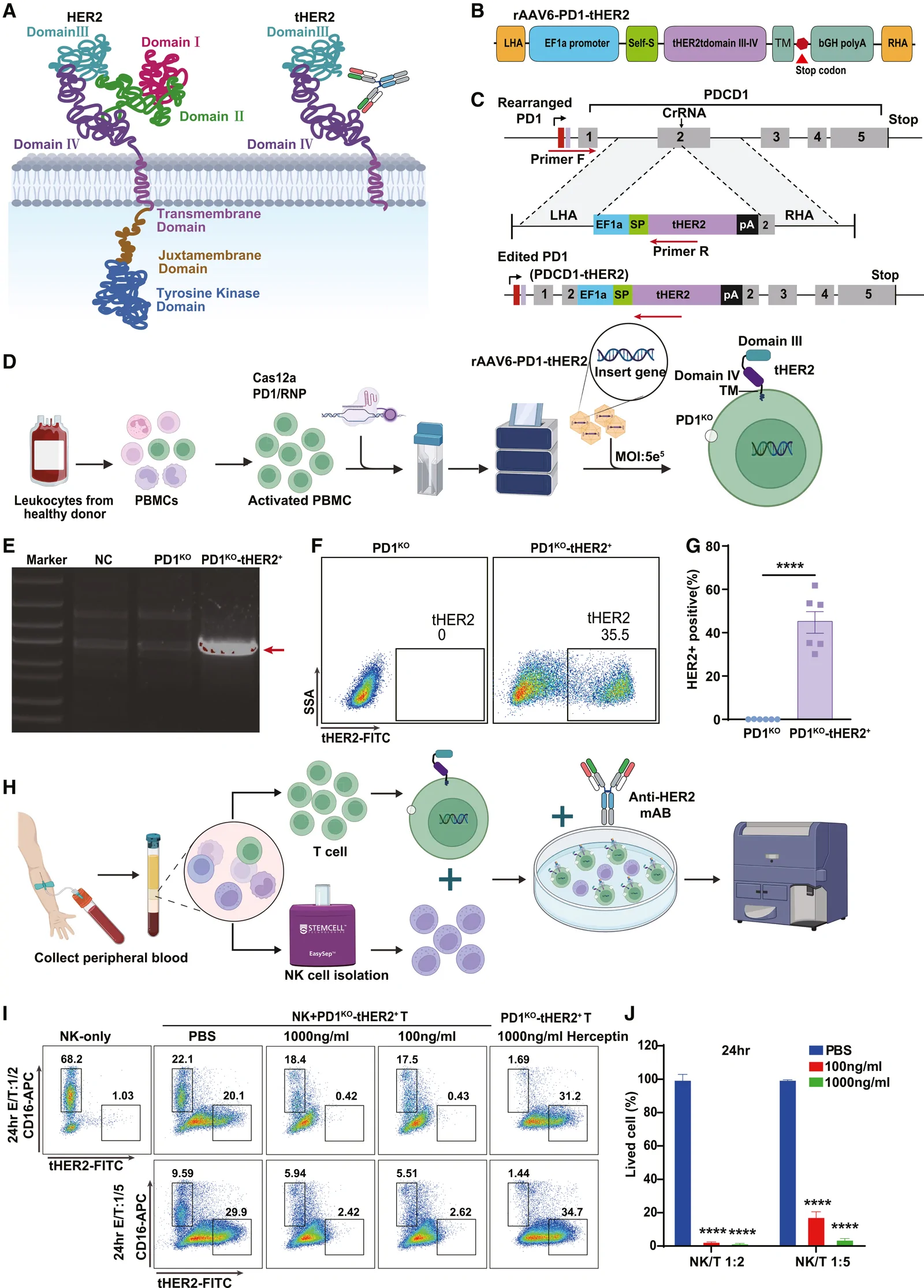
Herceptin-mediated depletion of tHER2-positive T cells *in vitro* (A) Diagram of HER2, tHER2, and anti-HER2 mono-antibody Herceptin binding to domain IV, domain I (red), domain II (green), domain III (wathet), and domain IV (purple), and the transmembrane and tyrosine kinase domain are indicated. (B) Conceptual of the recombinant adeno-associated virus serotype 6 (rAAV6) used for tHER2 expression cassette delivery into *PDCD1* locus. (C) Schematic representing the CRISPR-Cas12a-targeted tHER2 cassette integrated into the *PDCD1* locus. Top, *PDCD1* locus; middle, rAAV6 containing the tHER2 cassette flanked by homology arms; bottom, edited tHER2. (D) Schematic diagram of the strategy for site-specific inserting tHER2 cassette into the *PDCD1* cutting site on human primary T cells by using CRISPR-Cas12a/AAV6 electro delivery system. (E) PCR/agarose gel analysis of tHER2 site-specific integration into the *PDCD1* locus; the red arrow indicates the size of the tHER2 PCR product. (F) Human PBMCs engineered to express tHER2 cassette were assessed by flow cytometric dot-plots after 4 days of electro and AAV infection. (G) Statistical graph of HER2-positive surface expression in PBMCs from different human donors; *n* = 6 donors. Data shown as means ± SEM. *p* values were determined by two-tailed unpaired Student’s *t* test. ∗∗∗∗*p* < 0.0001. (H) Schematic diagram of the strategy to evaluate the ADCC effects by coculture of tHER2-positive T cells with isolated NK cells when adding anti-HER2 Herceptin antibody *in vitro*. (I) Representative flow cytometric dot-plots of the Herceptin-mediated NK and tHER2-positive T cell ADCC assay. (J) Frequency of live tHER2^+^ T cells in (I) after tHER2-positive T cells cocultured with NK cells with 100- and 1,000-ng/mL Herceptin for 24 h with NK:T ratio (1:2 and 1:5), quantified by FACS after 24 h. Data shown as means ± SEM. Data shown as means ± SEM; *p* values were determined using two-way ANOVA with Tukey’s correction for multiple comparisons (∗∗∗∗*p* < 0.0001). Schematics in (A)–(C) created using Adobe Illustrator. Schematics in (D) and (H) created using BioRender (https://Biorender.com).

### Herceptin-mediated depletion of tHER2-positive T cells *in vitro*

To evaluate the safety control capacity of the tHER2 switch in PD1^KO^-tHER2^+^ T cells *in vitro*, we established an ADCC model *in vitro*. Primary NK cells were isolated from fresh peripheral blood mononuclear cells (PBMCs) of healthy donors. Then, NK cells were cocultured with PD1^KO^-tHER2^+^ T cells derived from the same donor at various NK to T cell ratios (NK:T cell ratios) (Figure 1H).The specific cytotoxicity against tHER2-positive T cells was assessed 24 h after the addition of different concentrations of Herceptin to the coculture system. Flow cytometry analysis revealed that NK cell-mediated ADCC resulted in approximately 80% clearance of PD1^KO^-tHER2^+^ T cells at a lower NK:T ratio of 1:5 during treatment in the presence of a low concentration (100 ng/mL) of Herceptin and 95% clearance with a high concentration (1,000 ng/mL) at 24 h. Moreover, near-complete clearance, reaching up to 99%, was achieved at a higher NK:T ratio of 1:2 with both 100- and 1,000-ng/mL Herceptin at 24 h. In contrast, the PBS control group exhibited negligible cytotoxic effects (Figures 1I and 1J). Collectively, these results demonstrate that tHER2 functions as an effective safety switch, enabling potent and specific clearance of target cells upon antibody administration.

### Efficient integration of tHER2 into TCR-based CAR-T cells and maintenance of robust ADCC effect *in vivo*

To investigate whether the safety switch interferes with the cytotoxicity of CAR-T cells, we first constructed engineered T cells co-expressing tHER2 and the FMC63-scFv-based TCR-like CAR^45^ (termed FMC63-STAR^tHER2^) using the Cas12a/AAV editing system to simultaneously integrate tHER2 and the FMC63-STAR construct into the *PDCD1* and *TRAC* loci, respectively (Figures 2A and 2B). Flow cytometry analysis confirmed successful integration with *TRAC*-targeted FMC63-STAR gene insertion efficiency reaching 65%, and approximately 25% of the edited cells exhibited double-positive expression for both tHER2 and FMC63-STAR constructs (Figure 2C). The lower integration efficiency of FMC63-STAR in the control group was attributed to the lower MOI of the AAV. Cytotoxicity assays further demonstrated that the tHER2 safety switch did not impair the anti-tumor activity of FMC63-STAR-T cells (Figures S1A and S1B). Given that adverse events typically manifest several days after CAR-T cell infusion into patients, we next evaluated the Herceptin/ADCC-based suicide function *in vivo*. NOD/ShiLtJGpt-Prkdc^em26Cd52^Il2rg^em26Cd22^/Gpt (NCG) mice were intravenously infused with NK cells and beads enriched with tHER2-positive FMC63-STAR^tHER2^ T cells at an NK:T ratio of 1:1 (1 × 10^6^ NK cells vs. 1 × 10^6^ T cells). Herceptin at a dose of 10 mg/kg or PBS was administered intravenously the following day (Figure S2A). Flow cytometry analysis revealed that the population of HER2-positive T cells in peripheral blood was substantially eliminated as early as 16 h after Herceptin administration (Figures S2B and S2C). We further assessed the Herceptin/ADCC-based suicide function in FMC63-STAR-T cell therapy in mouse model of leukemia. NCG mice were first intravenously injected with 0.5 × 10^6^ CD19^+^ Raji-luc cells (Burkitt’s lymphoma cell line), and we next engineered FMC63-STAR^tHER2^ T cells and negative control (NC) T cells (tHER2-positive but STAR-negative T cells, tHER2^+^ T cells) infused 5 days later. A lower dose of NK cells (0.1 × 10^6^) was intravenously inoculated, and 10-mg/kg Herceptin or PBS was infused the next day (Figure 2D).Tumor progression and the pharmacokinetics of HER2-positive T cells in peripheral blood were monitored by bioluminescence imaging and flow cytometry at various time points (Figures 2E–2G). FMC63-STAR^tHER2^ T cells induced marked tumor regression as compared to the NC group (Figures 2E and 2F). Notably, the NK cells + Herceptin group exhibited significantly diminished anti-tumor efficacy following Herceptin treatment. Importantly, analysis of T cell proliferation in peripheral blood of NCG mice revealed that the number of tHER2-positive T cells decreased after NK cells + Herceptin treatment for 3 days and became nearly undetectable after 10 days of Herceptin administration (Figure 2G). Mice in the NK cells + Herceptin group also exhibited shortened survival, with all animals succumbing within 2 weeks after Herceptin treatment, whereas other treated groups exhibited better survival rates than NK cells + Herceptin group (Figure 2H). Collectively, these results demonstrate that NK cells combined with Herceptin treatment effectively eliminate tHER2-positive STAR-T cells *in vivo*. Thus, the tHER2 safety switch enables swift and efficient clearance upon administration of Herceptin, providing a valuable strategy for the rapid elimination of adoptive T cells when necessary.

**Figure 2:**
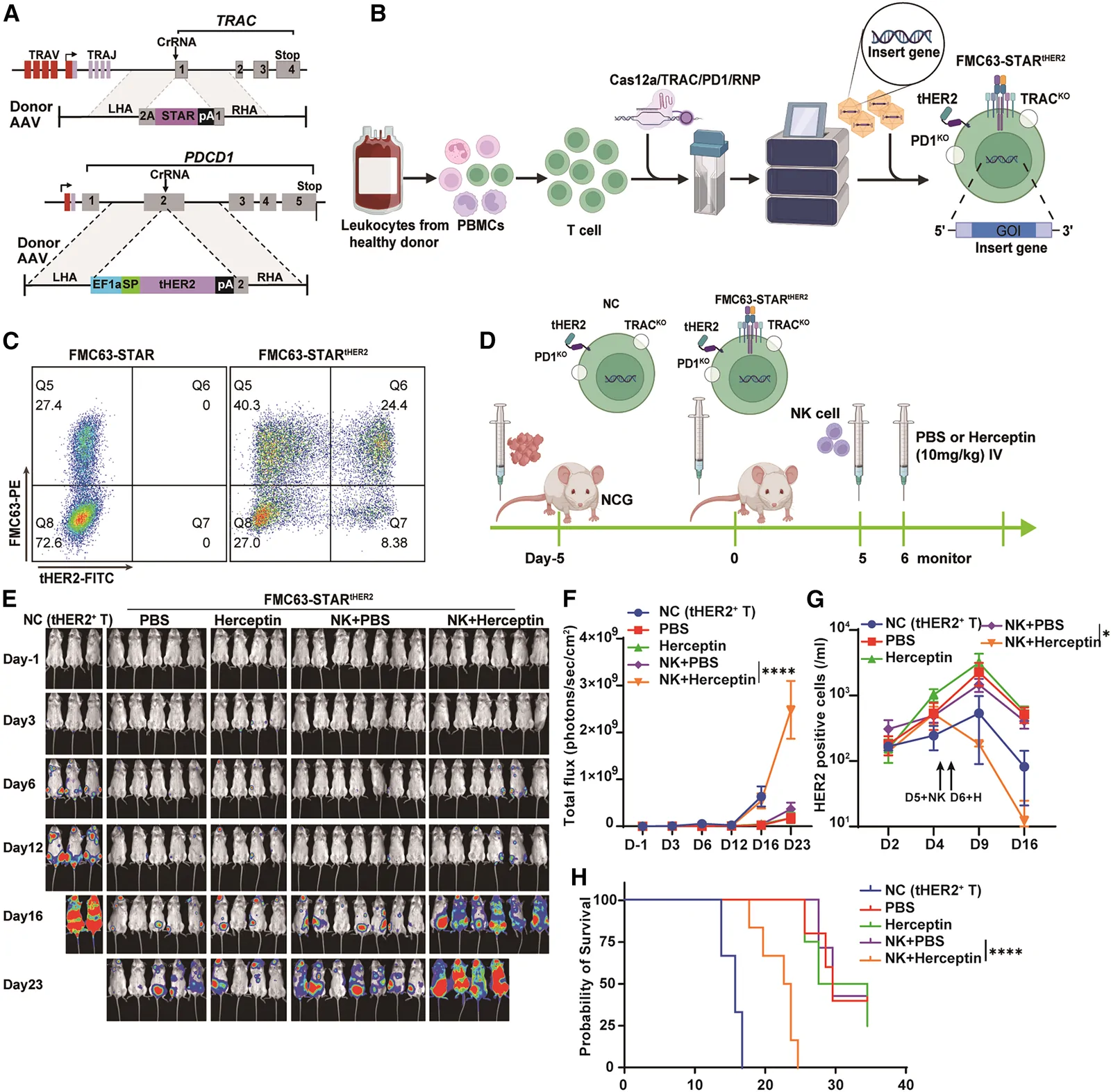
FMC63-STAR^tHER2^ T cells maintain robust suicide function *in vivo* (A) Schematic depicting the CRISPR-Cas12a-targeted tHER2 and FMC63-STAR gene integration into the *PDCD1* and *TRAC* locus. Top, *TRAC* locus and rAAV6 containing the STAR cassette flanked by homology arms; bottom, *PDCD1* locus and rAAV6 containing the tHER2 cassette flanked by homology arms. (B) Schematic diagram of the strategy to edit PD1^KO^-tHER2^+^ &TRAC^KO^-FMC63-STAR (FMC63-STAR^tHER2^) T cells by using CRISPR-Cas12a/rAAV6 electro delivery system. (C) Representative FMC63-STAR (PD1^KO^&TRAC^KO^ FMC63-STAR) and FMC63-STAR^tHER2^ flow cytometric dot-plots 4 days after electro and AAV infection. (D) Flow chart of the mouse *in vivo* experiment. 5 × 10^5^ CD19-expressing Raji-luc cells were injected into the tail vein of NCG mice for 5 days. Then NC (PD1^KO^&TRAC^KO^ tHER2^+^ FMC63-STAR non-integration T cell group, *n* = 3 mice) and FMC63-STAR^tHER2^ T cells were injected into mice (PBS, *n* = 5 mice; Herceptin, *n* = 5 mice; NK + PBS, *n* = 7 mice; NK + Herceptin, *n* = 6 mice). Subsequently, NK cells and Herceptin or PBS were administered via intravenous injection to NCG mice on day 5 and day 6 post T cell infusion. (E) Bioluminescence imaging of tumor cell growth following different treatments on the indicated days. (F) Time course of average tumor bioluminescence in NCG mice with various treatments over 23 days. Data shown as means ± SEM. (G) Analysis of tHER2-positive T cell proliferation and persistence in mouse peripheral blood samples, NC. Data shown as means ± SEM; *p* values determined by two-way ANOVA with Tukey’s correction for multiple comparisons comparing NK + Herceptin-treated group with NK + PBS group. (H) Kaplan-Meier survival curves of NCG mice from different groups. Data are analyzed by log rank (Mantel-Cox) test for survival analysis. Schematics in (A) created using Adobe Illustrator. Schematics in (B) and (D) created using BioRender (https://Biorender.com).

### Design of the dual-switch suicide system tHER2-TK

While the strong ADCC effect induced by tHER2 and Herceptin enables rapid T cell elimination, serving as a potential safeguard against secondary T cell malignancy, a gradual reduction in adoptive T cells is also desirable for long-term surveillance while minimizing adverse effects. Therefore, we investigated the feasibility of incorporating a slow-acting safety switch to achieve this goal. The herpes simplex virus type I-derived thymidine kinase (HSVTK) gene has been previously employed as an *in vivo* safety switch in donor T cell therapy for the treatment of recurrent malignancies and Epstein-Barr virus (EBV)-associated lymphoproliferation following hematopoietic stem cell transplantation.^46^^,^^47^^,^^48^^,^^49^ To enhance the controllability and safety of engineered cells, we combined tHER2 and HSVTK in tandem to construct a dual-switch suicide system (Figure 3A). We transfected 293T cells with tHER2 and tHER2-HSVTK (tHER2-TK) fusion constructs to confirm their expression (Figure 3B). After 20-h transfection, immunoblot analysis confirmed the successful expression of the tHER2 and tHER2-TK fusion proteins (Figures 3C and 3D). Subsequently, the tHER2-TK cassette was site-specifically integrated into the *PDCD1* locus of PBMCs with Cas12a/AAV gene editing system (Figures 3E and 3F). Flow cytometric analysis demonstrated stable expression of the tHER2-TK dual-switch suicide system on the T cell membrane, with a surface display efficiency of up to 50%, as compared with tHER2-only T cells (Figures 3G, 3H, 1F, and 1G). Collectively, these results indicate that the tHER2-TK dual-switch system was successfully integrated into the targeted genomic locus and highly expressed.

**Figure 3:**
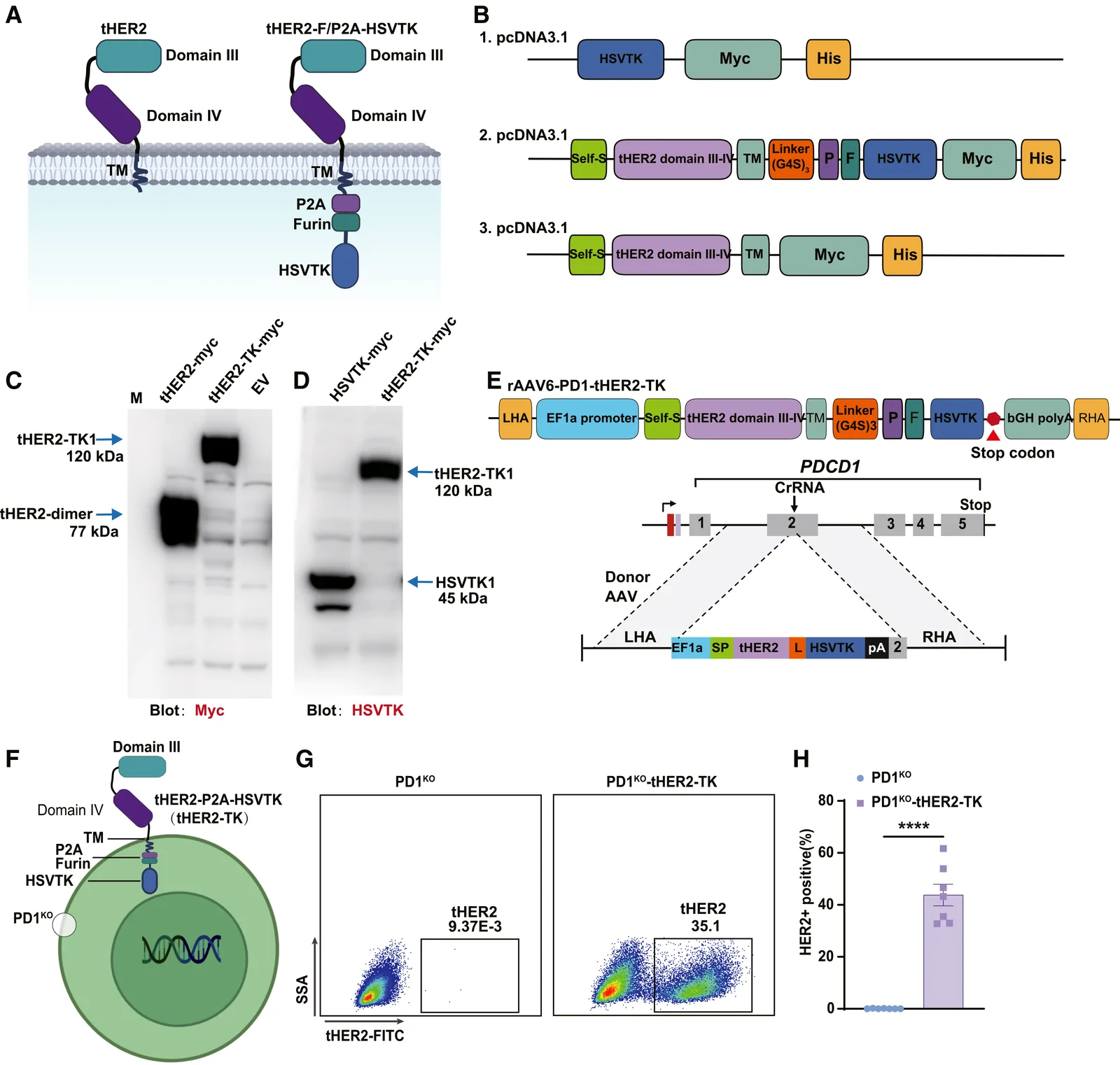
tHER2-HSVTK (tHER2-TK) dual-valency safety switch integrated into primary human T cells (A) Diagram of tHER2 single- and tHER2-TK double-target suicide gene safety switch. The HER2 domain III, domain IV, transmembrane (TM), Porcine Teschovirus-1 (P2A), Furin, and HSVTK (TK) domain are indicated. (B) Architecture of envelope (EV), tHER2, and tHER2-TK constructs into pcDNA3.1-Myc vector. (C and D) Western blot confirming expression of tHER2-TK and HSVTK by detecting fusion Myc tag (C) or HSVTK (D). (E) Schematic diagram of the strategy for site-specific inserting tHER2-TK cassette into the *PDCD1* cutting site on human primary T cells by using CRISPR-Cas12a/AAV6 electro delivery system. (F) Schematic diagram of the successfully edited tHER2-TK T cells. (G) Flow cytometric dot-plots of engineered tHER2-TK human T cells after 4 days of electro and AAV infection. (H) Statistical graph of HER2-positive surface expression in PBMCs from different human donors; *n* = 7 donors. Data shown as means ± SEM. *p* values were determined by two-tailed unpaired Student’s *t* test. ∗∗∗∗*p* < 0.0001. Schematics in (A), (B), and (E) created using Adobe Illustrator. Schematics in (F) created using BioRender (https://Biorender.com).

### Functional dual-switch safety system exhibited differential clearance kinetics to editing T cells

We further investigated the functionality of the combinatorial safety switch tHER2-TK in FMC63-STAR-T cells (FMC63-STAR^tHER2-TK^) (Figure 4A). Flow cytometry analysis confirmed that approximately 20% of the edited cells were double positive for both tHER2-TK and FMC63-STAR constructs (Figure 4B). To examine whether tHER2-TK expression disturbs the effector function of FMC63-STAR-T cells, we cocultured FMC63-STAR^tHER2-TK^ T cells with CD19^+^ Raji-luc tumor cells. Functionally, FMC63-STAR^tHER2-TK^ T cells exhibited comparable cytotoxicity to that of control FMC63-STAR-T cells (Figure 4C). To evaluate the regulatory capacity of the dual-switch system in response to Herceptin and GCV *in vitro*, we cocultured the primary NK cells with FMC63-STAR^tHER2-TK^ T cells. Specific cytotoxicity against tHER2-positive T cells was monitored at different time points (8–96 h) and across a range of Herceptin concentrations to assess safety-switch functionality. The results demonstrated that, at a higher NK:T ratio (1:1 to 1:2), Herceptin treatment at concentrations greater than 50 ng/mL led to approximately 70% clearance of FMC63-STAR^tHER2-TK^ T cells within 16 h and nearly complete elimination (approximately 98% by day 4) (Figures 4D and S3A). Furthermore, the clearance efficiency of FMC63-STAR^tHER2-TK^ T cells was significantly enhanced with increasing dosage and treatment duration. At concentrations above 50 ng/mL, Herceptin achieved nearly complete eradication of FMC63-STAR^tHER2-TK^ cells *in vitro* following 96 h of coculture with NK cells at a 1:20 NK:T ratio (Figures 4D and S3A).

**Figure 4:**
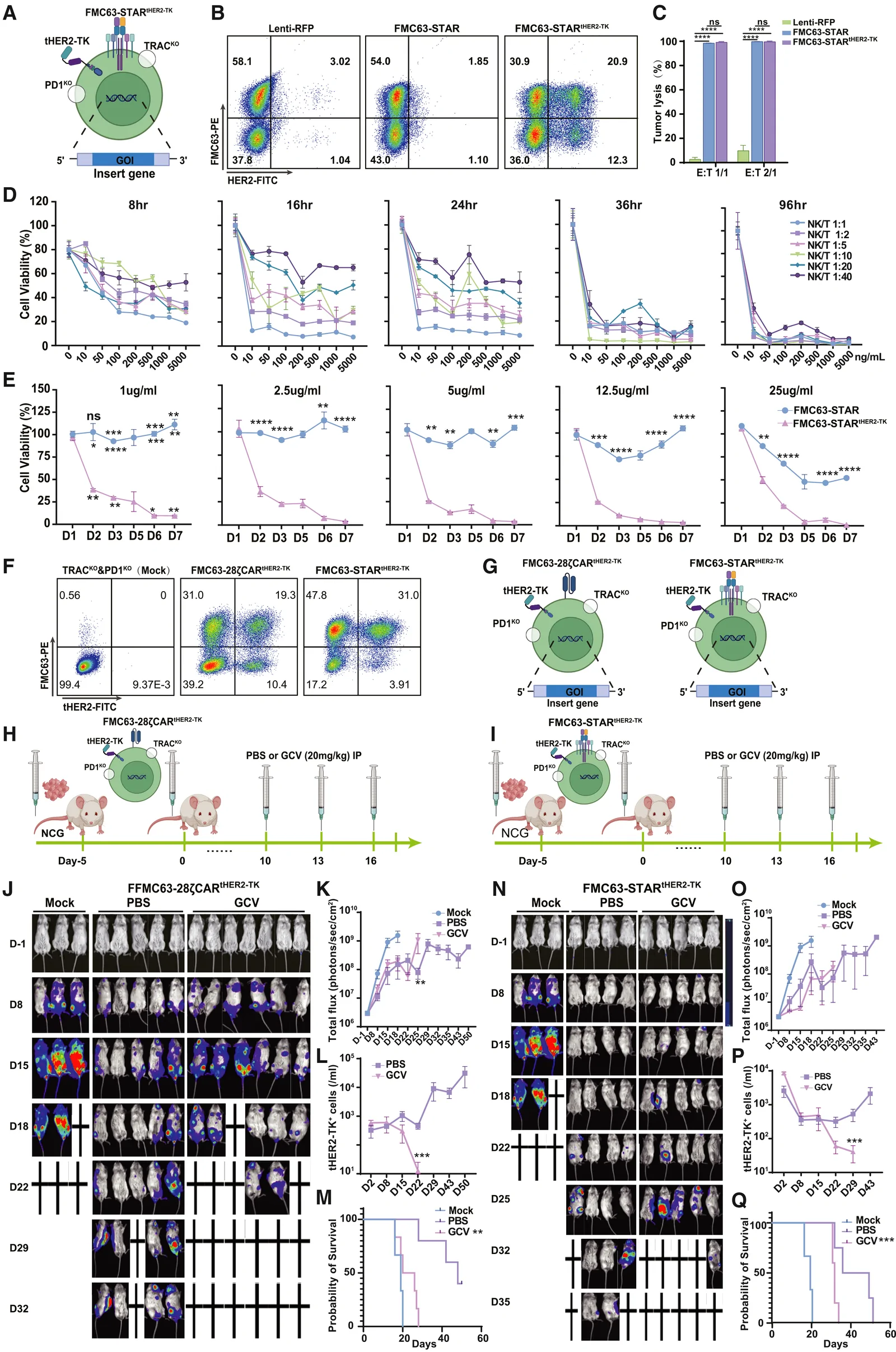
tHER2-TK confers robust Herceptin/ADCC and HSVTK/GCV-based suicide gene function (A) Schematic diagram of the tHER2-TK and FMC63-STAR site-specific integration into the *PDCD1* and *TRAC* disrupt locus. (B) Flow cytometric dot-plots of Lenti-RFP, FMC63-STAR, and FMC63-STAR^tHER2-TK^ T cells. (C) Cytotoxicity of FMC63-STAR and FMC63-STAR^tHER2-TK^ T cells toward CD19-expressing Raji-luc target cells after 18-h coculture by luciferase-based tumor-killing assay. (D) A Herceptin dose- and time-escalation experiment was conducted to assess the different NK:T ratios of NK cell against FMC63-STAR^tHER2-TK^ T cell cytotoxicity. Data shown as means ± SEM. (E) Titration of GCV concentrations and assessment of their cytotoxic effects on tHER2-TK-positive T cells over time. Cells were treated with indicated concentrations of GCV, and cell viability was measured on days 1, 2, 3, 5, 6, and 7. Data shown as means ± SEM; *p* values were determined using two-way ANOVA with Tukey’s correction for multiple comparisons (∗*p* < 0.05, ∗∗*p* < 0.01, ∗∗∗*p* < 0.001, and ∗∗∗∗*p* < 0.0001; ns, not significant). (F) Flow cytometric dot-plots of PD1^KO^&TRAC^KO^ (mock), FMC63-28ζCAR^tHER2-TK^, and FMC63-STAR^tHER2-TK^ T cells. (G) Schematic diagram of FMC63-28ζCAR^tHER2-TK^ and FMC63-STAR^tHER2-TK^ T cells. (H and I) Flow chart of the mouse *in vivo* experiment. 5 × 10^5^ CD19-expressing Raji-luc cells were injected into the tail vein of NCG mice for 5 days. Then mock (*n* = 3 mice), FMC63-28ζCAR^tHER2-TK^ (PBS, *n* = 5 mice; GCV, *n* = 7 mice) (H) or FMC63-STAR^tHER2-TK^ (PBS, *n* = 4 mice; GCV, *n* = 5 mice) (I) T cells were injected intravenously into mice. Subsequently, GCV was administered via intraperitoneal injection to NCG mice on day10, 13, and 16 post T cell infusion. (J and N) Bioluminescence imaging of tumor cell growth following different treatments on the indicated days. (K and O) Time course of average tumor bioluminescence in NCG mice with various treatments over 50 days. Data shown as means ± SEM. (L and P) Analysis of tHER2-TK-positive T cell proliferation and persistence in mouse peripheral blood samples. Data shown as means ± SEM; *p* values determined by two-way ANOVA with Tukey’s correction for multiple comparisons; compare GCV-treated group with PBS group. (M and Q) Kaplan-Meier survival curves of NCG mice from different groups. Data are analyzed by log rank (Mantel-Cox) test for survival analysis. Data shown as means ± SEM (∗∗*p* < 0.01 and ∗∗∗*p* < 0.001). Schematics in (A) and (G–(I) created using BioRender (https://Biorender.com).

We next titrated the GCV concentrations and treatment durations to evaluate the safety-switch functionality mediated by the HSVTK/GCV system. The viability of FMC63-STAR-T^tHER2-TK^ cells was assessed following treatment with GCV at concentrations ranging from 1 μg/mL (low) to 25 μg/mL (high) at various time points. These results indicated that even a low concentration of GCV (1 μg/mL) was sufficient to induce significant cell death following 2 days of exposure. Prolonged exposure led to a drastic reduction in cell viability, falling below 5% by day 6. In contrast, higher concentrations of GCV (e.g., 12.5 and 25 μg/mL) elicited potent and rapid cytotoxicity, achieving near-complete elimination of tHER2-TK-positive cells within 5 days (Figures 4E and S3B). Although all tested GCV concentrations eventually achieved nearly 100% clearance of tHER2-TK-expressing T cells by day 7, the cytotoxic effects were clearly dose dependent, with higher concentrations of GCV causing severe cell cytotoxicity (Figures 4E and S3B). Collectively, these findings demonstrated that the tHER2-TK dual-switch system can effectively eliminate tHER2-TK-positive cells in a dose- and time-dependent manner through their ADCC or GCV pathway individually. Notably, the tHER2/ADCC regulatory system exhibited more rapid and efficient target cell killing and clearance in the early phase, whereas the HSVTK/GCV system displayed a more gradual and sustained regulatory profile for engineered T cells.

### FMC63-28ζCAR^tHER2-TK^ and STAR^tHER2-TK^ confer robust HSVTK/GCV-based suicide function *in vivo*

To evaluate the *in vivo* safety-switch efficacy of tHER2-TK regulatory system in different CAR-T cell architectures, we first generated FMC63-28ζCAR^tHER2-TK^ and FMC3-STAR^tHER2-TK^ T cells, achieving integration efficiencies of approximately 20% and 30%, respectively, using the Cas12a/AAV editing system (Figures 4F and 4G; the gating strategy is shown in Figure S5B). Gene schematics of FMC63-STAR and FMC63-28ζCAR are respectively shown in Figures S2D and S2E. To establish a xenograft model, NCG mice were infused with CD19^+^ Raji-luc tumor cells. Five days post inoculation, the mice received injections of either FMC63-28ζCAR^tHER2-TK^, FMC63-STAR^tHER2-TK^ T cells or negative control T cells (PD1^KO^&TRAC^KO^, mock) according to their respective groups. Subsequently, mice received intraperitoneal administration of GCV on days 10, 13, and 16 post T cell infusion, and the dynamics of tHER2-positive T cell populations and tumor progression were monitored (Figures 4H and 4I). GCV treatment resulted in significantly accelerated tumor progression and increased bioluminescence intensity compared with the PBS-treated groups in both the FMC63-28ζCAR^tHER2-TK^- and FMC63-STAR^tHER2-TK^-infused mice model (Figures 4J, 4K, 4N, and 4O). No significant difference was observed in the peripheral blood counts of tHER2-TK-positive T cells between groups prior to GCV administration. However, following several rounds of GCV treatment, a sharp reduction in tHER2-TK-positive cells was observed in the GCV-treated groups, with HER2-positive cells being nearly completely cleared by day 12 after the first GCV administration (Figures 4L and 4P). These results suggest that GCV treatment leads to gradual clearance of the infused FMC63-28ζCAR^tHER2-TK^ or FMC63-STAR^tHER2-TK^ T cells, thereby abrogating the tumor-killing activity of CAR- or STAR-T cells *in vivo*. Mice in the GCV-treated CAR and STAR groups also exhibited shortened survival, with all animals succumbing within 2–3 weeks after GCV treatment, whereas PBS-treated mice survived for more than 45 days (Figures 4M and 4Q). Collectively, these results demonstrate that the tHER2-TK dual-switch system exhibits robust dual molecular functionality both *in vitro* and *in vivo*.

### The safety switch does not compromise the effector function of engineered T cells

Next, we examined the effector function of safety-switch-containing adoptive T cells compared to conventional engineered T cells both *in vitro* and *in vivo* studies. Site-specific integration of the safety switch in *PDCD1* locus does not compromise the tumor-killing functionality of FMC63-28ζCAR cells (Figure S4A). *In vitro* cytotoxicity assays demonstrated that CAR-T cells harboring the tHER2-TK safety switch exhibited comparable tumoricidal activity to that of conventional CAR-T cells at lower effector-to-target (E/T) ratios of 1:10 and 1:20 (Figure S4A). Furthermore, *in vivo* anti-tumor experiments in NCG mice confirmed the site-specific integration of the safety switch into CAR-T cells, which did not impair their tumor-eliminating capacity (Figures S4B and S4C). Importantly, a single administration of Herceptin at a lower dose was sufficient to achieve rapid clearance of tHER2-TK-positive T cells *in vivo* (Figures S4B–S4E). Collectively, these findings indicate that incorporation of the suicide gene switch does not compromise the effector functions of CAR-T cells, and no significant functional deficits were observed.

### tHER2 switch enables highly efficient enrichment of tHER2-positive cells using magnetic beads

Highly efficient isolation of engineered T cells is a critical step during T cell preparation prior to infusion, ensuring that any adverse events triggered by the infused cells can be effectively controlled. We therefore tested whether the safety-switch construct could also serve as an effective tool for isolating engineered T cells. T cell-expressed tHER2-TK construct could be identified by flow cytometry, indicating that flow cytometry sorting could be employed to purify engineered T cells. Meanwhile, immunomagnetic-bead-based sorting enables efficient and specific enrichment of target cells. We therefore designed antibodies targeting tHER2 specifically, a full-length anti-HER2 mAb, and combined them with magnetic beads for T cell isolation (Figure 5A). The production and specificity of the purified antibodies were evaluated by flow cytometry (Figure 5B). Engineered cells were stained with either a commercial anti-HER2 fluorescein isothiocyanate (FITC)-conjugated antibody or our produced anti-HER2 mAb. Flow cytometry results demonstrated that anti-HER2 mAb exhibited high binding specificity to FMC63-STAR^tHER2-TK^ T cells, achieving tHER2-positive detection rates of approximately 50%, which was consistent with the results obtained using the commercial anti-HER2-FITC antibody (positive control [PC]) (Figures 5C and 5D). Subsequently, we assessed the effectiveness of antibodies for magnetic-bead sorting. The purified anti-HER2 mAb was chemically conjugated to nano-magnetic beads (supplied by Yifei(Hangzhou)Bio-tech) and used for the purification of tHER2-TK-positive CAR- or STAR-T cells (Figures 5E and 5F). Using these antibody-coupled magnetic beads, we magnetically sorted FMC63-28ζCAR^tHER2-TK^ or FMC63-STAR^tHER2-TK^ T cells, with tHER2-positive cells sorted by fluorescence-activated cell sorting (FACS) serving as a PC. The results demonstrated that magnetic sorting achieved high purification, yielding approximately 90% purity for both FMC63-28ζCAR^tHER2-TK^ and FMC63-STAR^tHER2-TK^ T cells, which was consistent with the purity obtained via FACS (Figures 5G and 5H). The gating strategy is shown in Figure S5C. These findings indicate that immunomagnetic beads enable efficient and specific enrichment of tHER2-positive T cells. Collectively, the tHER2-TK dual-switch system enables efficient suicide function individually via ADCC or the chemical drug GCV (Figure 5I), providing a valuable strategy for the elimination of adoptively transferred T cells when necessary.

**Figure 5:**
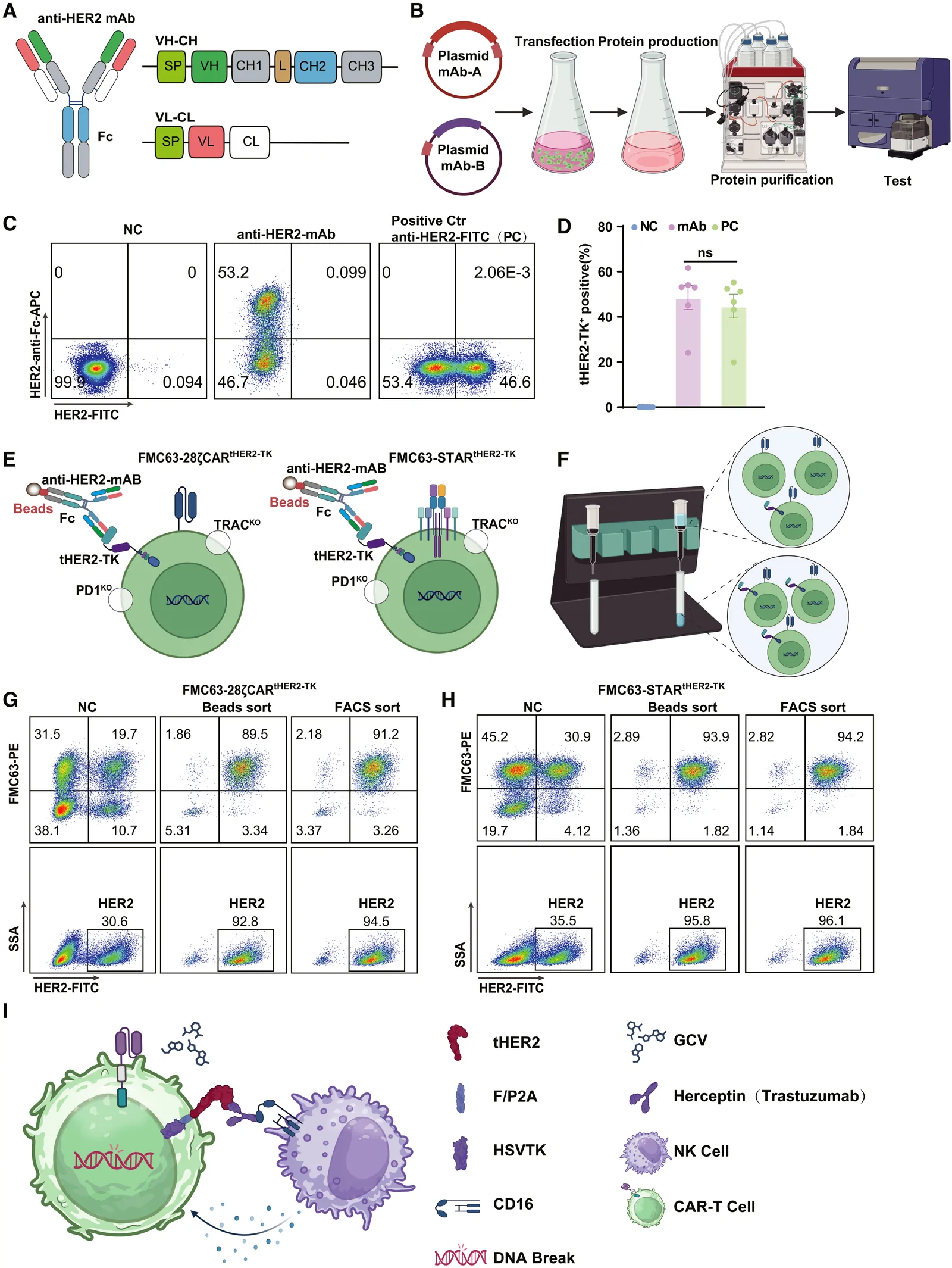
Anti-HER2 mAb-conjugated magnetic beads highly isolate tHER2-TK-positive cells (A) Scheme of the anti-HER2 mAb and plasmids for production the antibody protein. (B) Scheme of anti-HER2 mAb production in 293F cells and purification with high-performance fast protein liquid chromatography (FPLC) systems such as ÄKTA and testing with FACS staining. (C) Flow cytometric dot-plots of anti-HER2 mAb with anti-Fc-conjugated APC second antibody-stained tHER2-TK-positive T cells and commercial anti-HER2 antibody (FITC) as a-positive control (PC). (D) Percentage of tHER2-TK positive in (C), quantified by FACS after staining. *n* = 6 donors. Data shown as means ± SEM; two-tailed unpaired Student’s *t* test. ns, not significant. (E) Cartoon illustrating the specific binding of magnetic-bead-conjugated HER2 mAb to HER2-positive T cells. (F) Schematic for the isolation of gene-edited tHER2-positive T cells. (G and H) Flow cytometric dot-plots of the isolated FMC63-28CAR^tHER2-TK^ (G) and FMC63-STAR^tHER2-TK^ (H) T cells, cells sorted by magnetic beads (MACS), or flow cytometry sorting (FACS); sorted cells were cultured for 2 days and then stained for flow cytometric analysis. (G) and (H) are a representative results from one of three independent experiments. (I) Schematic diagram of the dual-target tHER2-TK safety-switch function. Schematics in (A) created using Adobe Illustrator. Schematics in (B), (E), (F), and (I) created using BioRender (https://Biorender.com).

## Discussion

Insertion of CAR constructs into T cells or gene editing also carry risks of insertional mutagenesis, off-target effects, and oncogenic transformation.^50^^,^^51^ Recently, a serious adverse effect of uncontrolled clonal T cell proliferation due to insertional mutagenesis of retroviral vector was reported in the X-linked severe combined immunodeficiency (X-SCID) gene therapy clinical trial.^52^ These and other toxicities are potentially reversible if a conditional safety switch is incorporated. In this study, we developed an optimized dual-valency safety switch comprising a truncated cell-surface HER2 domain in combination with an intracellular HSVTK domain. This system can be used for time-efficient *in vitro* purification, *in vivo* tracking, and rate-tunable elimination of transferred T cells, and it was designed to enhance the safety and controllability of next-generation T cell therapies.

The dual-switch system allows for independent or simultaneous activation via two clinically tractable drugs: the mAb Herceptin (which triggers NK cells mediate ADCC)^53^ and the small-molecule drug GCV, which activates the HSVTK pathway.^23^^,^^54^ The tHER2 component enables rapid and efficient target cell clearance, whereas the HSVTK component mediates delayed clearance that is more dependent on the GCV dosage. The advantage of combining these two systems lies in the ability to selectively achieve either rapid or gradual clearance depending on the specific clinical context, thereby maximizing the reduction of toxic side effects without compromising the therapeutic efficacy of T cells. The Herceptin/ADCC safety switch offers a rapid-antibody-dependent clearance mechanism, which is ideal for addressing acute toxicities. In contrast, the HSVTK/GCV pathway provides a molecular-level safety switch that acts on DNA level and remains effective even in immune-privileged sites where antibody penetration or effector cell engagement may be limited. The consistent observation that GCV-mediated clearance, while highly effective, exhibits slightly slower kinetics compared with the rapid ADCC response further underscores the complementary nature of these two mechanisms. A significant technical advancement presented here is the efficient, targeted integration of the tandem tHER2-TK expression cassette into the *PDCD1* locus of primary human T cells using a combined Cas12a/RNP and rAAV6 donor template delivery strategy. This approach serves a dual purpose: it disrupts the *PDCD1* gene, potentially reducing T cell exhaustion and enhancing persistence,^55^ while simultaneously placing the safety switch under exogenous transcriptional regulation only. This strategy is superior to random viral integration, which carries risks of insertional mutagenesis and variegated transgene expression.^56^ Furthermore, this dual-suicide platform also possesses cell-purification functionality using anti-HER2 antibody-conjugated magnetic beads, enabling the isolation of high-purity tHER2-positive cell populations. This purification capability is essential for ensuring a uniform T cell product with reliable safety regulation.

Cetuximab is a clinically approved antibody that is associated with significant adverse effects—most notably skin and subcutaneous tissue disorders (reported in approximately 20.88% of patients), as well as infusion-related reactions.^57^ tHER2-based switch achieves rapid and efficient clearance, as demonstrated by near-complete T cell elimination within 24 h. This rapid depletion capability is particularly advantageous for managing acute toxicities such as CRS or on-target off-tumor effects, where delayed elimination could compromise patient safety. Although trastuzumab is associated with well-documented cardiotoxicity (including arrhythmia, cardiomyopathy, and heart failure), these risks are established in the setting of chronic, repeated-dose administration for HER2-positive breast cancer therapy—typically administered weekly or every 3 weeks over many months.^58^^,^^59^ In contrast, our safety switch is designed for low-dose, single-administration use only when rapid elimination of adoptively transferred cells is clinically required. This fundamental difference in dosing regimen substantially mitigates the cardiotoxicity concern, as trastuzumab-mediated cardiac dysfunction is not strictly dose dependent but, rather, is related to sustained HER2 blockade. Importantly, clinical evidence demonstrates that trastuzumab cardiotoxicity is often reversible and can be managed with appropriate monitoring.^60^ In the models tested here, the tHER2/trastuzumab system produced rapid depletion of engineered T cells following trastuzumab administration. The TK safety switch in our system serves an auxiliary function. The primary rapid-elimination mechanism is mediated by the HER2/trastuzumab switch, which is designed for low-dose, single-administration use to achieve rapid and efficient ablation of the majority of adoptively transferred cells in acute-toxicity scenarios. The TK component is not intended for rapid, complete depletion; rather, its role is complementary—to gradually eliminate residual cells. Its “delayed” kinetics are functionally advantageous for maintaining long-term surveillance while enabling eventual clearance. The dual-switch strategy provides redundant, complementary control that a single-switch system cannot offer, enhancing overall safety and flexibility.

Despite these promising results, our study has limitations. The research was conducted in immunodeficient mouse models,^61^ which precludes the assessment of potential immunogenicity of the transgenic proteins (tHER2 and HSVTK) in a fully functional human immune system. HSVTK is a viral protein derived from herpes simplex virus, and its non-human origin inherently carries the risk of eliciting both humoral and cellular immune responses in immunocompetent host, which could trigger immune-mediated clearance of engineered cells in immunocompetent hosts, potentially rendering TK incorporation disadvantageous compared with tHER2 alone. We therefore acknowledge that the auxiliary designation of TK may impose an additional immunogenic burden in immunocompetent hosts. To address this concern in future studies, systematic evaluation of TK immunogenicity should be conducted in immunocompetent or humanized mouse models with comprehensive immune monitoring. Concurrently, ongoing efforts in our laboratory are directed toward deimmunizing the HSVTK protein through epitope engineering while preserving its enzymatic activity for GCV-mediated killing. From a clinical perspective, we envision a risk-stratified application of our safety platform. The tHER2-alone switch offers a rapid T cell ablation as the HER2-mediated ADCC effect lies in its dual mechanism of immune synergy and precise recognition; the side effect is usually lower in the commonly used humanized antibodies in clinical practice. However, the dual-switch tHER2-TK system retains unique value in high-risk settings, such as allogeneic T cell therapies or high-dose engineered cell products—where fail-safe redundancy is paramount. Thus, the platform provides a flexible, context-adaptive solution rather than a one-size-fits-all strategy. Furthermore, while we demonstrated high efficiency, the technical complexity—involving gene editing, viral transduction, and magnetic-bead-based isolation—presents manufacturing challenges that must be addressed to optimize broad clinical feasibility. In the context of the evolving field, our tHER2-TK system compares favorably with other safety switches. It moves beyond first-generation single-mechanism switches (e.g., CD20/Rituximab or HSVTK alone) by offering dual-valency safety control.^54^^,^^62^ While technologies such as inducible caspase-9 (iCas9) offer ultra-rapid kinetics, they often rely on fully synthetic ligands.^25^^,^^26^ In contrast, our system leverages two clinically approved therapeutic drugs with well-established safety profiles, which may potentially accelerate regulatory approval.

In conclusion, we have engineered a versatile and highly efficient dual-targeting safety-switch system that addresses key limitations of previous platforms. The tHER2-TK switch provides flexible, rapid, and dual-valency safety control over engineered T cells, supported by a scalable manufacturing process for generating high-purity products. This strategy significantly enhances the safety profile of ACT by mitigating the risks of acute toxicity and long-term oncogenic potential through the use of either mAbs or small-molecule drugs at any stage of treatment. Moreover, it provides a safeguard mechanism that enables timely intervention and improved safety without compromising therapeutic efficacy, thereby facilitating safer and more controllable CAR-T-based cancer immunotherapy. Future work will focus on engineering the HSVTK protein to mitigate its immunogenic potential, evaluating safety switch in more advanced, immunocompetent models and streamlining the manufacturing process to pave the way for clinical translation, ultimately aiming to unlock the full therapeutic potential of ACT with greater confidence and safety.

## Materials and methods

### Experimental model and study participant details

#### Cell lines and culture

HEK293 T cells for AAV packaging were purchased from American Type Culture Collection (ATCC). Lenti-X-293T cells for lentivirus packaging were purchased from Takara Biomedical Technology. 293T cells were cultured in DMEM with 10% fetal bovine serum (FBS) with 1% penicillin-streptomycin (Sigma, V900929), 1 mM sodium pyruvate (Gibco, 11360-070), 1% GutaMAX (Gibco, 35050-061), and 1% minimum essential medium (MEM) non-essential amino acids (Gibco, 11140-050). The Raji tumor cells were lentivirally transduced to express firefly luciferase GFP under control of the EF-1α promoter. All cells were cultured at 37°C and 5% CO_2_ incubator. Cells were regularly tested for *Mycoplasma* using PCR and found to be negative.

#### Production of fusion protein

For the anti-HER2 (clone 24D2)-derived antibody, the variable region of light chain (VL), variable region of heavy chain (VH), and human IgG1 constant region were synthesized. The anti-HER2 mono-antibody sequence in this study was derived from trastuzumab, utilizing the light chain and heavy chain regions sourced. All these constructs were further cloned into the pcDNA3.1 vector. Then, vectors were transfected into 293-F cells. Supernatants were collected and purified using protein A sepharose according to manufacturer instructions.

#### *In vitro* human T cell isolation and culture

PBMCs from healthy donor Leukopaks (Shanghai OriBiotech) were isolated by Ficoll-Plaque PLUS gradient centrifugation. T cells were isolated by negative selection beads and stimulated with precoated anti-human CD3 (clone OKT3) (BioLegend, 317326), mouse anti-human CD28 (clone CD28.2) (BD, 555725), and human fibronectin (Roche, 10838039001). T cells were cultured in X-VIVO15 medium supplemented with 10% heat-inactivated FBS, 1% penicillin-streptomycin (Sigma, V900929), 1 mM sodium pyruvate (Gibco, 11360-070), 1% GutaMAX (Gibco, 35050-061), and 1% MEM non-essential amino acids (Gibco, 11140-050) and recombinant human IL-2 (200 IU/mL).

#### CRISPR editing of primary human T cells

The CRISPR editing process was achieved by electroporation of RNP and addition of AAV as a knockin donor vehicle to the cell medium. For each electroporation condition, 37.5 pmol of each CRISPR crRNA mixed with 15.8 pmol (2.5 μg) of Alt-R A.s. Cas12a (Cpf1) Ultra (IDT, 10001273) in a total volume of 5 μL of nuclease free Opti-medium for at least 10 min at 37°C. Before electroporation, T cells were centrifuged at 90 × *g* for 5 min, resuspended at 1 × 10^6^ T cells in 20 μL of P3 buffer (Lonza, V4XP-3032), and mixed with 5 μL of the Cas12a RNP. The mixture was nucleofected in a 16-well cuvette with a Nucleofector X-Unit (Lonza, AAF-1003X) using pulse code EO115. After electroporation, 40 μL of prewarmed T cell medium was added to each well and the cuvette strip was placed at a 37°C cell incubator for 10 min. Then, the cells were transferred to 48-well plate with 500 μL of T cell medium. The AAV-carrying knockin donor was added to the medium within 30 min with an MOI of 5E^5^ genome copies after the electroporation process. AAV packaging and titer titration were performed by PackGene Biotech (Guangzhou, China) as a custom service. The AAV titer was determined and adjusted to be 1E^13^ genome copies/mL. T cell medium was changed every other day until for sorting or functional assays on days 10 or later after initial activation.

For simultaneously multiple genes editing, all the crRNAs mixed with scaled-up Cas12a nuclease were nucleofected into T cells instead of separately incubating each RNP and combining together.

TRAC-As Cas12a crRNA sequence:

5′-UAAUUUCUACUCUUGUAGAUGAGUCUCUCAGCUGGUACAC-3′

PD1-As Cas12a crRNA sequence:

5′-UAAUUUCUACUCUUGUAGAUgcacgaagcucuccgaugug-3′

PCR was performed using the 2× Hieff PCR Master Mix (With Dye), and the following primer sets: PD1, forward 5′-gtgaaggctcttagtaggaaatc-3′, PD1/tHER2 reverse 5′-ctcctggatattggcactg -3′.

#### Primary NK cell isolation and coculture

PBMCs from healthy donors were isolated by Ficoll-Plaque PLUS gradient centrifugation. NK cells were isolated by using EasySep Human NK Cell Isolation Kit (catalog #17955, STEMCELL, negative selection). Isolated NK cells were cocultured with tHER2 or tHER2-TK edited T cells from same donor supplement with trastuzumab (MCE, HY-P9907) and cultured in X-VIVO15 medium supplemented with 10% heat-inactivated FBS, 1% penicillin-streptomycin (Sigma, V900929), 1 mM sodium pyruvate (Gibco, 11360-070), 1% GutaMAX (Gibco, 35050-061), and 1% MEM non-essential amino acids (Gibco, 11140-050) and recombinant human IL-2 (200 IU/mL).

The NK cells used in the in *vivo* experiments were derived from umbilical cord blood (UCB) from (Shanghai OriBiotech). In brief, isolated CD34+ UCB cells were differentiated and expanded using NK cell differentiation medium. This medium consisted of the same basal medium as used for the CD34 expansion step supplemented with 10% (horse serum) HS, the low-dose cytokine cocktail, and a new high-dose cytokine cocktail consisting of 20-ng/mL IL-7, 22-ng/mL SCF, 1,000-U/mL IL-2 (Proleukin; Chiron, München, Germany), and 20-ng/mL IL-15 (CellGenix).^63^^,^^64^ Medium was refreshed twice a week from day 14 onward. Flow cytometric analysis revealed that the CD56^+^CD3^−^ NK cells accounted for more than 96% of the total cell population.

#### Homology-directed repair template design

All the insertion genes (synthesized by Genscript Biotechnological, Nan Jing, China) flanked with 5′ homology and 3′ homology arms was cloned into the site between two inverted terminal repeats (ITRs) on rAAV6 construct. The insertion genes were either separated by furin-2A sequence or EF1α promoter sequence from the endogenous gene locus. A polyadenylate terminator was added after the stop codon. Homology arms were about 300 base pairs in length. Unless specified in figure legends, all FMC63-28ζCAR or FMC63-STAR constructs were adopted from previous published design in our lab, in which TCRα and TCRβ constant regions are murine chains with a disulfide-bond modification.

#### Lentivirus production and transduction

Lentiviruses were packaged with the packaging and envelop plasmids psPAX2 and pMD2.G and the shuttle vectors carrying genes of interest or RFP in Lenti-X-293T cells. The culture-medium supernatant was collected at 24 and 48 h after culture medium was changed. The virus was concentrated via high-speed centrifugation at 20,000 rpm for 2 h at 4°C and then suspended with RPMI-1640 medium (final volume/solute volume = 1:100) then stored at −80°C. T cell transduction was performed in 48-well plates. After 24–48 h of stimulation, T cells were transduced with concentrated lentivirus at 1,500 rpm for 90 min at 32°C.

#### Mouse xenograft models

Female NCG mice aged 6–8 weeks were obtained from GemPharmatech and were maintained and treated in compliance with Institutional Animal Care and Use Committee (IACUC)-approved research protocols. Intravenous Raji-luc cells and T cells were injected individually via tail vein in 200 μL of PBS. All the injection timing is noted in the figure legends. Tumor progression was quantified with bioluminescence signal. Mouse peripheral blood samples were collected through orbital sinus. For measurement of blood volume and flow cytometric analysis, the volume (V) of blood collected from each mouse was measured using a 100-μL pipette. Then the blood was treated with red blood cell (RBC) lysis buffer and washed with PBS, followed by flow cytometry antibody staining. Each stained blood sample was resuspended in 200 μL of FACS buffer, analyzed by flow cytometry, and a uniform volume of 100 μL was collected from each sample. The number of T cells expressing different markers was calculated. Taking HER2-positive cells as an example, the total number of HER2-positive cells in 1 mL of blood = (total number of HER2-positive cells in collected 100 μL sample × 2)/measured blood sample volume V × 1,000 μL. No randomization or blinding methods were used.

#### Cytotoxicity assays

Sorted FMC63-STAR^,^ or FMC63-28ζCAR T cells were cocultured with targeted tumor cell (e.g., CD19-Raji-luc) at the indicated effector/target ratios (E/T). Cytotoxicity was measured by luciferase reporter assay. After coculture for 18 h in 96-well plates, cells in each well were lysed with 100 μL of lysis buffer (Yeasen, 11401-A) at room temperature for 20 min and then centrifuged at 4,000 rpm for 20 min at 4°C. 20 μL of the supernatant was taken to the luciferase plate, 50 μL of luciferin (11401-B) was added to each well, and the luciferase intensity was measured immediately using GloMax NAVIGATOR (Promega) according to the manufacturer’s instructions.

#### Magnetic beads and biomolecule coupling method

For magnetic beads pretreatment, transfer 100 μL of the beads into a 1.5-mL EP (Eppendorf) tube. Separate the beads magnetically and discard the supernatant. Add 200 μL of MEST (0.1 M Morpholinoethanesulfonic Acid, pH 6.0, 0.05% Tween 20, pH 6.0, 0.05% Tween 20) solution, separate magnetically, and discard the supernatant. Repeat this step 2–3 times. For EDC (1-Ethyl-3- (3-dimethylaminopropyl) carbodiimide hydrochloride) activation, quickly add 100 μL of EDC solution (10 mg/mL EDC, 0.1 M MES, pH 6.0, 0.05% Tween 20) and 100 μL of NHS solution (10 mg/mL N-hydroxysulfosuccinimide) to the pre-treated beads. Mix by inversion on a vertical rotator to keep the beads in suspension, and activate at 25 °C for 1 h. For bead conjugation, separate the beads magnetically and discard the supernatant. Immediately add the biological ligand (50–200 μg). Mix by inversion on a vertical rotator to keep the beads suspended. Conjugate at 25 °C for 2 h, or conjugate at 25 °C for 1 h followed by overnight incubation at 4 °C. For blocking: separate the beads magnetically and discard the supernatant. Resuspend the beads in 200–500 μL of blocking buffer. Mix by inversion on a vertical rotator to keep the beads suspended. Block at 25°C for 2–4 h or overnight at 4°C. Storage separate the beads magnetically and discard the supernatant. Wash the conjugated product three times with 200 μL of PBS Buffer. Add an appropriate volume of storage solution, mix well to resuspend the beads, and store at 4°C.

#### Immunomagnetic cell separation

Resuspend the cells to be separated and purified in PBS containing 1% fetal bovine serum, count and adjust the cell density. Transfer 1 × 10^8^ cells into a 50-mL centrifuge tube and centrifuge at 1,500 rpm for 5 min. Discard the supernatant. Resuspend the cells in 90 μL of buffer per 1 × 10^7^ cells, then add 100 μL of immunomagnetic beads and incubate at 4°C for 20 min. Washing: after incubation, add 29 mL of buffer and centrifuge at 300 × *g* for 5 min. Discard the supernatant and resuspend the cells in 500 μL of buffer (maximum cell number for resuspension: 1 × 10^8^ cells per 500 μL). Separation using a magnetic separation column: (1) remove the column from its packaging, place it on the magnetic rack, and prime the column (add 3 mL of buffer to prime each column before use). (2) After the buffer has stopped dripping, load the sample cells onto the column for separation. (3) Wash the column three times with 3 mL of buffer each time. (4) After the buffer has stopped dripping, remove the column from the magnetic rack, place it over a 15-mL centrifuge tube, add 5 mL of buffer, and promptly push out the plunger to collect the cells retained on the column into the centrifuge tube. Centrifuge the 15-mL centrifuge tube containing the enriched cell suspension at 2,000 rpm for 5 min. Resuspend the cells in 5 mL of buffer, count the cells, and proceed with subsequent experiments. Note that the buffer is PBS containing 1% FBS.

#### Calculation of recovery and viability

Count the enriched cells. Take 1 × 10^6^ cells and stained them with BD Horizon Fixable Viability Stain 510 and anti-HER2 flow cytometry antibody. Based on the positivity rate of BD Horizon Fixable Viability Stain 510 staining, calculate the viability of the sorted cells (100% − m% of V510). Calculation of cell enrichment rate: percentage of HER2-positive cells × total number of cells recovered/number of HER2-positive cells before magnetic bead separation.

#### Western blot

T cells were collected in lysis buffer (50 mM HEPES [pH 7.4], 150 mM NaCl, 1% NP-40, and 1 mM EDTA) containing 1 mM sodium orthovanadate, 1 mM sodium fluoride, 1 mM phenylmethylsulfonyl fluoride (PMSF), and a protease inhibitor mixture (Roche). Total cell lysates or nuclear extractions were subjected to SDS-polyacrylamide gel electrophoresis (PAGE) and transferred onto a polyvinylidene difluoride membrane (Millipore). The membrane was sequentially probed with primary antibodies (4°C overnight, antibody dilution 1:1,000) and horseradish peroxidase-conjugated secondary antibodies (room temperature for 1 h, antibody dilution 1:10,000). Enhanced chemiluminescence substrates (Pierce) were used to visualize the specific bands on the membrane. Primary antibodies used included thymidine kinase mAb (Santa Cruz Biotechnology, sc-377211) and Myc-Tag antibody (Cell Signaling, 2272S).

#### Flow cytometry

The fixable viability dye eFlour 506 was used to label the viable cells (Invitrogen, 65-0866-14). Anti-FMC63-STAR and FMC63-28ζCAR expression were measured by biotin-anti-FMC63-scFv (Bioswan, 500014) and followed by Streptavidin-APC (BioLegend, 405207) or Streptavidin-PE (BioLegend, 405203). For T cell phenotyping, the following antibodies were used: CD3-FITC (BioLegend, 300406), CD8-PerCpCy5.5 (BioLegend, 344710), CD8-BV605 (BioLegend, 564116), CD4-APC (BioLegend, 300514), human CD45-BV421 (BioLegend, 368522), human CD45-PerCpCy5.5 (BioLegend, 368504), human TCRα/β-BV421 (BioLegend, 306721), PD1-BV421 (BioLegend, 329920), anti-human CD340 (erbB2/HER-2) (BioLegend, anti-human CD340 (erbB2/HER-2) antibody), anti-human CD56 antibody, clone HCD56 (catalog #60021), and APC anti-human IgG Fc antibody (BioLegend, 410712).

#### Statistical analysis

Statistical analysis was performed using GraphPad Prism 9 software. Groups were compared using the Welch’s two-sample *t* test for parametric data or the Mann-Whitney test for non-parametric data. We used Welch’s correction, as the variances were not equal. Statistical comparisons between multiple groups were determined by one-way analysis of variance (ANOVA) or two-way ANOVA, and Tukey’s multiple comparison test was used. *p* < 0.05 was considered to be statistically significant. The statistical test used for each figure is described in the corresponding figure legend.

## Data and code availability

All relevant data are available from the authors.

## Acknowledgments

This work was supported by the 10.13039/100031910Changping Laboratory (2026C-02-01), 10.13039/501100001809National Natural Science Foundation of China (82294366062), and Noncommunicable Chronic Diseases-10.13039/501100018537National Science and Technology Major Project (2023ZD0520200).

## Author contributions

Experiment design, H.L., J.W., and X.L.; data analysis assistance, H.L., J.W., and H.W.; sample and information collection, H.L.; validation experiments, H.L.; writing – original draft, H.L.; writing – review and editing, H.L., H.W., and J.W.; scientific input and editing, H.L., J.W., and X.L. All authors discussed and approved the manuscript.

## Declaration of interests

One independent patent application has been filed to protect the technology described in this study, in which H.L., H.W., J.W., and X.L. are stated as inventors.

## Supplemental information

Document S1. Figures S1–S5

Document S2. Article plus supplemental information
