# Supplementary material for: A dual-switch safety system enables safety controllability and high-efficiency enrichment for T cell immunotherapy
Source: Mol Ther Adv. 2026 Oct 14;34(4):201865. doi: 10.1016/j.omta.2026.201865 (PMC13742281; doi:10.1016/j.omta.2026.201865)
Supplement: Supplementary file 1 [file mmc1.pdf]

OMTA, Volume *34*

Supplemental information

A dual-switch safety system enables safety controllability and high-efficiency enrichment for T cell immunotherapy

Hongtao Liu, Huimin Wang, Bo Hou, Wenxin Zhou, Hanfei Zhu, Xin Lin, and Jiasheng Wang

SUPPLEMENTAL MATERIALS

Supplemental figures

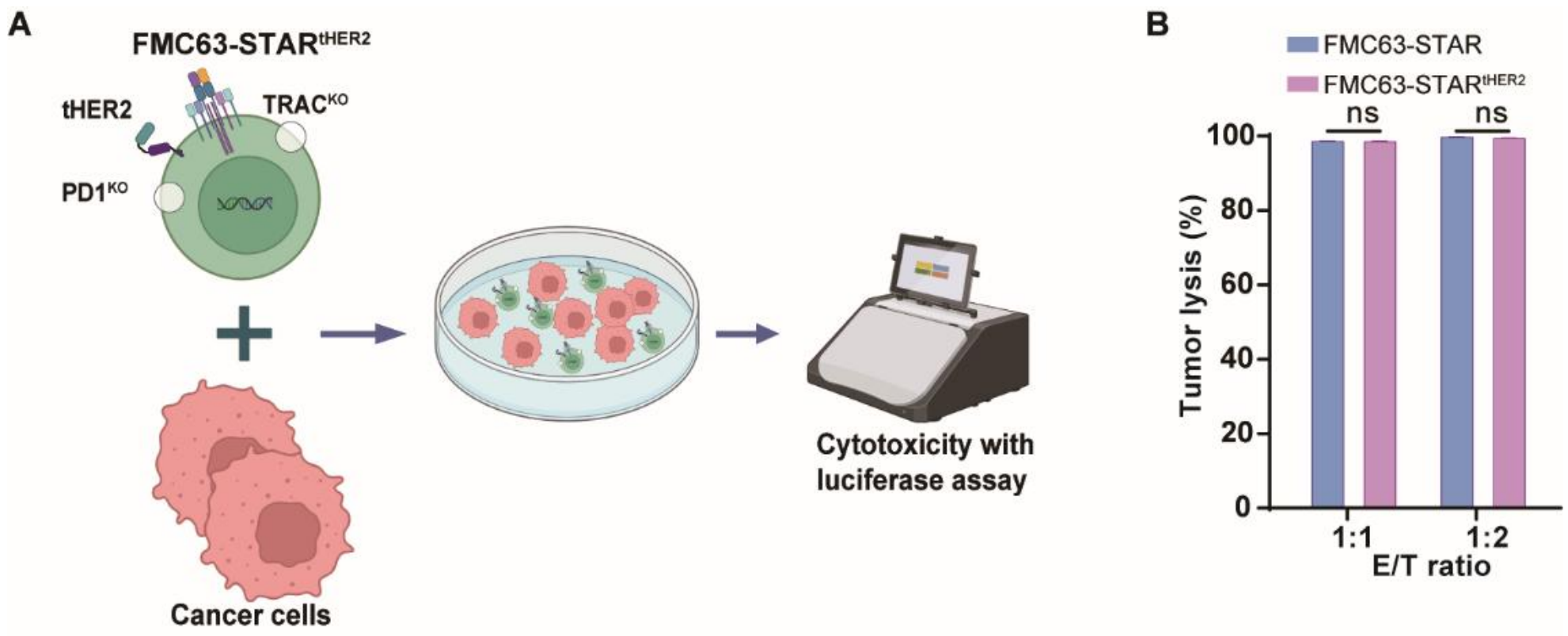

Figure S1: Edited FMC63-STAR^{tHER2} T cells remain intact effector function

(A) Schematic diagram of the coculture tumor killing assay. (B) Cytotoxicity of FMC63-STAR and FMC63-STAR^{tHER2} T cells toward CD19 expressing Raji-luc target cells after 18-hour coculture by Luciferase-based tumor killing assay. Data shown as means ± SEM; Two-tailed unpaired Student's *t* test. ns,not significant. Schematics in **A** created using BioRender (https://Biorender.com).

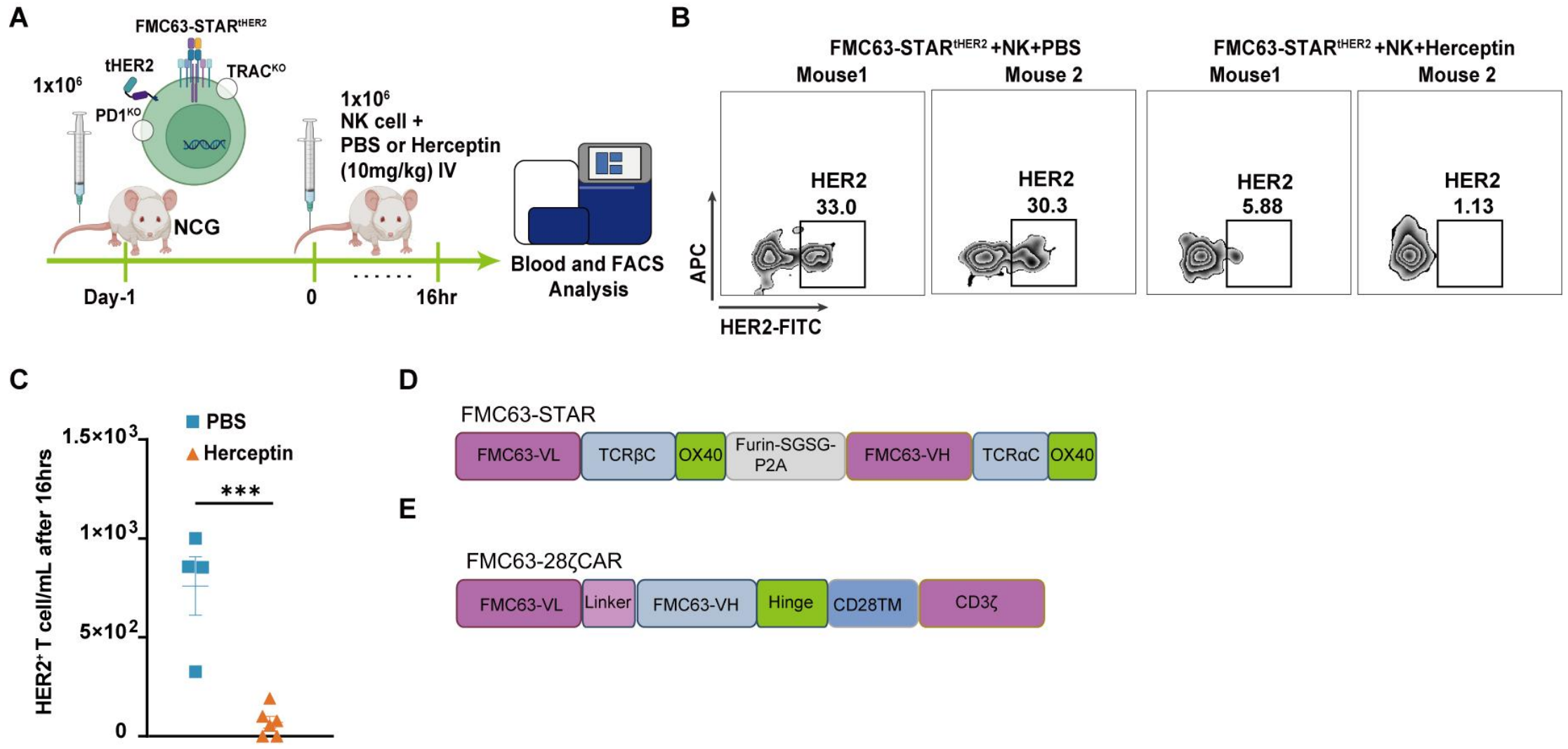

Figure S2: tHER2 cells maitain swift Herceptin/ADCC-based suicide function in vivo

(A) Flow chart of the mouse in vivo experiment. 1×10^6 FMC63^{tHER2} T cells were injected into the tail vein of NCG mice for 1 days. Subsequently, 1×10^6 NK cells and Herceptin or PBS was intravenously infused at another day, next Flow cytometry analysis the population of tHER2-positive T cells in peripheral blood 16 hours later. (B) Flow cytometric dot-plots analysis of HER2 positive T cells survival in mouse peripheral blood samples. (C) Statistical graph of HER2 positive T cells in mouse peripheral blood samples in (B). (D-E) Gene schematics of FMC63-STAR and FMC63-28ζCAR that carry polycistronically expressed. Data shown as means ± SEM. P values were determined by Two-tailed unpaired Student's t-test. ****P < 0.0001. Schematics in **A** created using BioRender (https://Biorender.com). Schematics in **D**, **E** created using Adobe Illustrator.

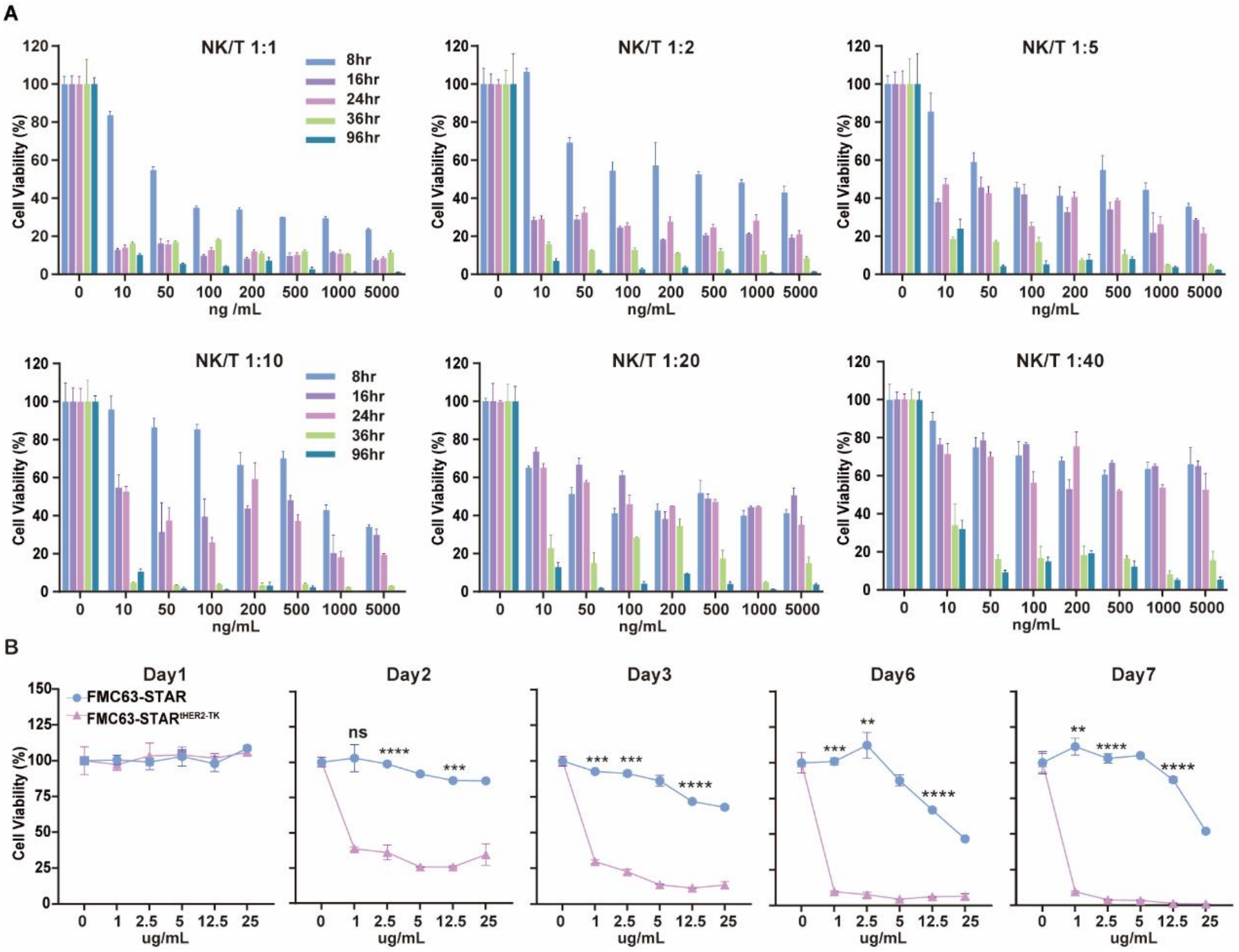

Figure S3: Dual-valency suicide gene HER2t-HSVTK confer robust Herceptin/ADCC and HSVTK/GCV-based suicide gene function

(A) A Herceptin dose- and time-escalation experiment was conducted to assess the different E/T ratio of NK cell against FMC63-STAR$^{\text{tHER2-TK}}$ T cells cytotoxicity. Data shown as means ± SEM. (B) Titration of different GCV concentrations to the HSVTK effects of killing tHER2-TK-positive T cells in day1, 2, 3, 5, 6 and 7. Data shown as means ± SEM; P values were determined using two-way ANOVA with Tukey's correction for multiple comparisons (*P < 0.05, **P < 0.01, ***P < 0.001, and ****P < 0.0001; ns, not significant).

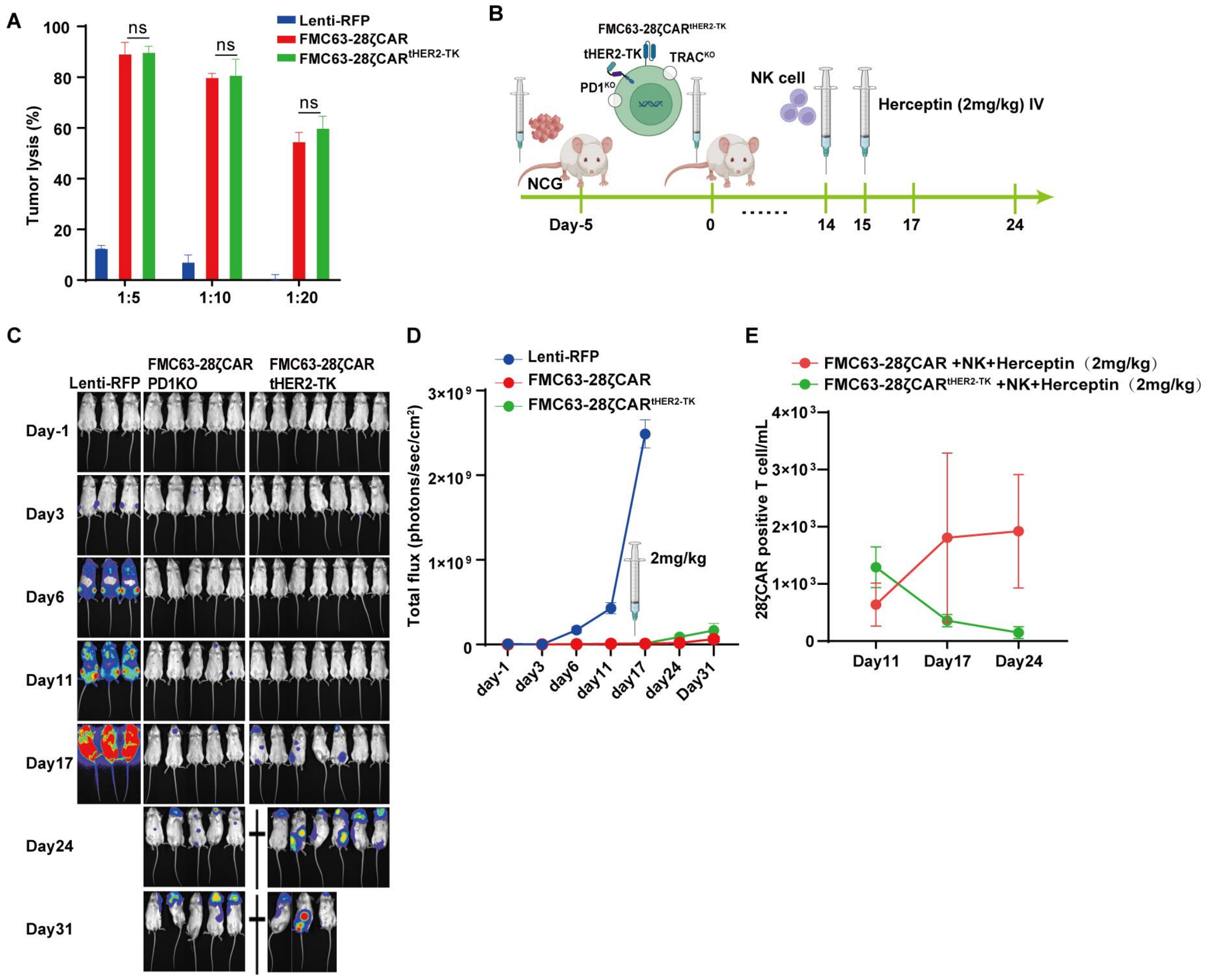

Figure S4: CAR-T cell effector function following safety switch integration

(A) Cytotoxicity of Lenti-RFP control, FMC63-28ζCAR and FMC63-28ζCAR $^{tHER2-TK}$ T cells toward CD19 expressing Raji-luc target cells after 18-hour coculture by Luciferase-based tumor killing assay at lower E/T ratio (1:5,1:10,1:20). (B) Flow chart of the mouse *in vivo* experiment. 5×10^5 CD19 expressing Raji-luc cells were injected into the tail vein of NCG mice for 5 days. Then Lenti-RFP, FMC63-28ζCAR and FMC63-28ζCAR $^{tHER2-TK}$ T cells were injected into mice. Subsequently, NK cells, and Herceptin was administered via intravenous injection to NCG mice on day 14 and day 15 post T-cell infusion. (C) Bioluminescence imaging of tumor cell growth following different treatments on the indicated days. Lenti-RFP, n=3 mice; FMC63-28ζCAR, n=5 mice and FMC63-28ζCAR $^{tHER2-TK}$, n=7 mice. (D) Time course of average tumor bioluminescence in NCG mice with various treatments during 31 days. Data shown as means ± SEM. (E) Analysis of FMC63-28ζCAR positive T cell proliferation and persistence in mouse peripheral blood samples. Data shown as means ± SEM; P values

determined by two-way ANOVA with Tukey's correction for multiple comparisons. Two-tailed unpaired Student's *t* test. ns,not significant. Schematics in **B** created using BioRender (https://Biorender.com).

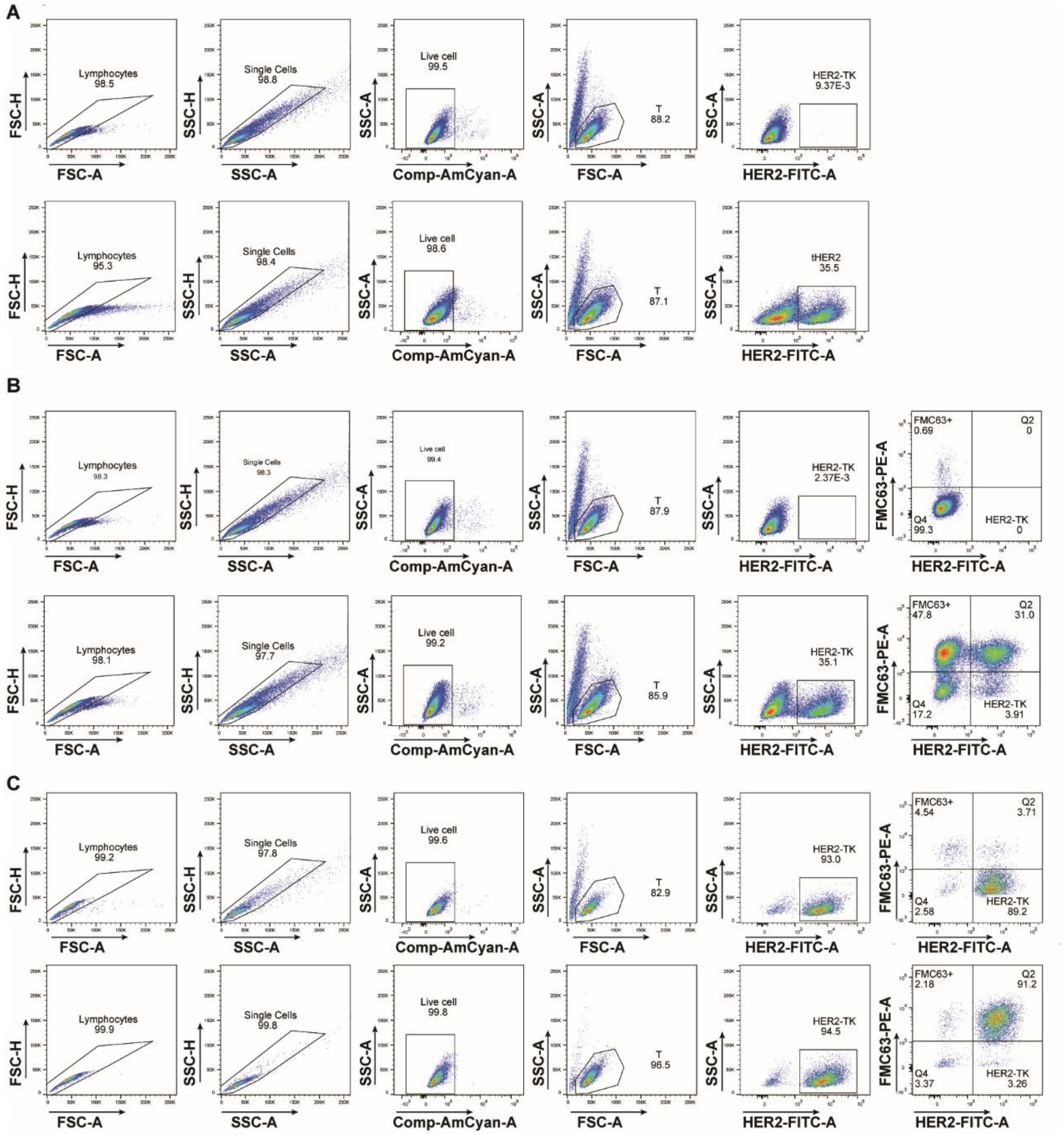

Figure S5: Gating strategies and examples of the Flow data

(A) Gating strategy and examples for detecting the surface display of tHER2 on the T cell membrane, as shown in Figure 1F. (B) Gating strategy and examples for detecting the surface display of STAR receptors and tHER2 on the T cell membrane, as shown in Figure 2C, 4B, and 4F. (C) Gating strategy and examples for detecting the purified FMC63-STAR$^{tHER2\text{-}TK}$ T cell, as shown in Figure 5G and 5H.
